# Supplementary material for: Familial atrial fibrillation mutation M1875T-SCN5A increases early sodium current and dampens the effect of flecainide
Source: Europace. 2022 Dec 12;25(3):1152–61. doi: 10.1093/europace/euac218 (PMC10062360; doi:10.1093/europace/euac218)
Supplement: euac218_Supplementary_Data [file euac218_supplementary_data.docx]

**Supplemental Figures**


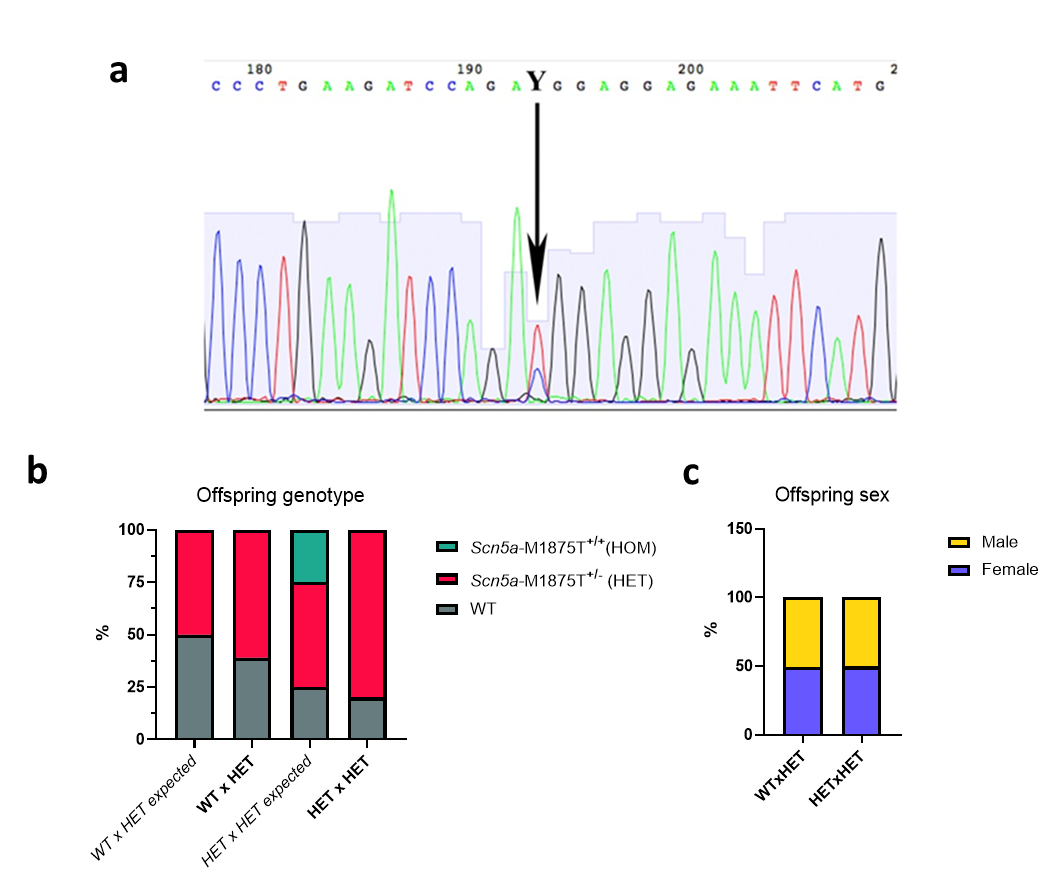


**Figure S1: Sequencing analysis of F1 offspring, and subsequent offspring genotype and sex ratios.** **a)** Sequencing analysis of the heterozygous F1 offspring animal number 8 (Figure 1b). Position of the T-C mutation, which corresponds to M1875(7)T amino acid exchange in the protein sequence, is labeled by an arrow. **b)** Observed offspring genotype and **c)** sex distribution, analysed from 90 litters (901 animals in total), pairing with WTxHET or HETxHET animals. WT, wildtype; HET, heterozygous; HOM, homozygous.

**
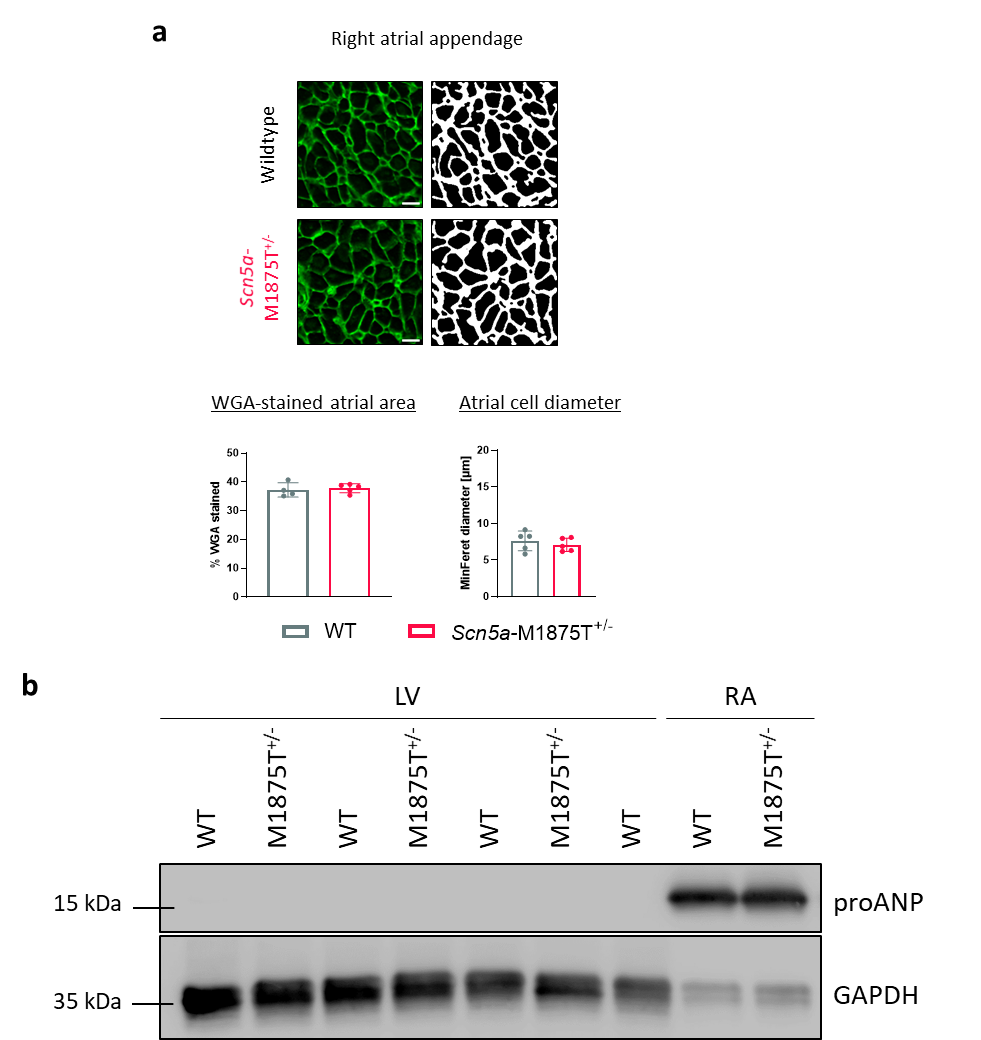
**

**Figure S2: Histological phenotyping of wildtype and *Scn5a*-M1875T^+/-^ right atria and ventriclular and right atrial proANP protein level assessment**

**a)** Exemplary immunofluorescence images from atrial regions of interest (ROI) of wheat germ agglutinin (WGA, green) staining and corresponding binary images for quantification in left atrial (LA) appendage and LA posterior wall from wildtype (WT, left) and *Scn5a*-M1875T^+/-^ (right) mice. Scale bars represent 10 µm. Graphs show quantification of WGA-stained atrial area and atrial cell diameters from transverse sections as depicted above. Neither parameter was affected by the point mutation at young adult age (WGA-stained atrial area quantified from LA appendage: N=6 hearts and 22/21 individual ROI per group; Atrial cell diameter quantified from N=6 hearts and 8548/11883 individual cells per group. Data are presented as mean ± SD. **b)** Immunoblot probing for pro-ANP in left ventricular (LV) as well as right atrial (RA) tissue. GAPDH expression serves as loading reference with reduced loading for the atrial samples. There was no ventricular signal for proANP, excluding overt heart failure.


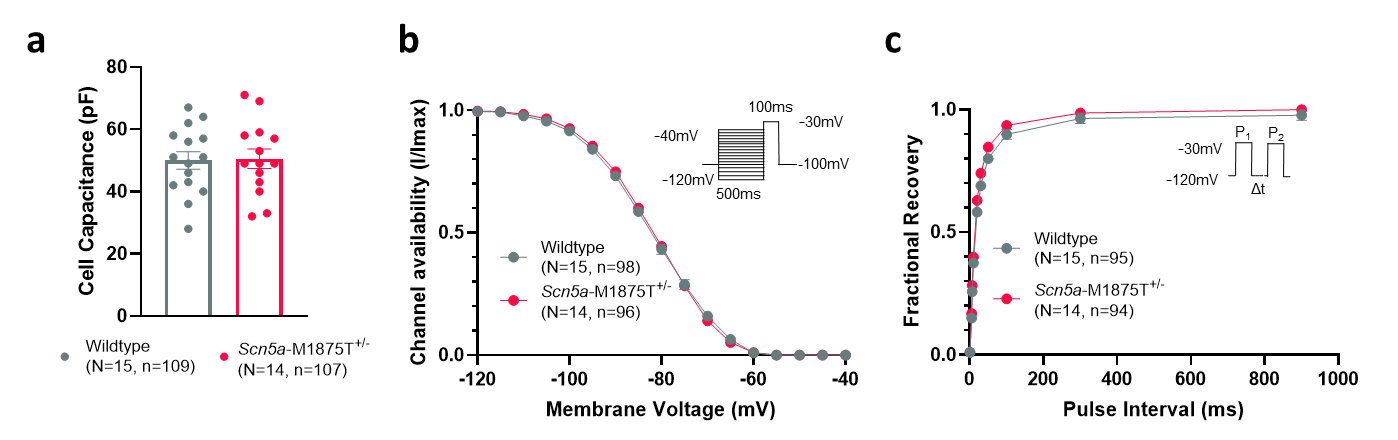


**Figure S3: Measurements of cell capacitance, voltage-dependent inactivation and time-dependent recovery kinetics of I_Na_ using patch clamp electrophysiology. a)** Cell capacitance measurements exclude overt hypertrophy, **b)** voltage-dependent inactivation and **c)** time-dependent recovery kinetics of I_Na_ in isolated left atrial cardiomyocytes from wildtype and *Scn5a*-M1875T^+/-^ mice.


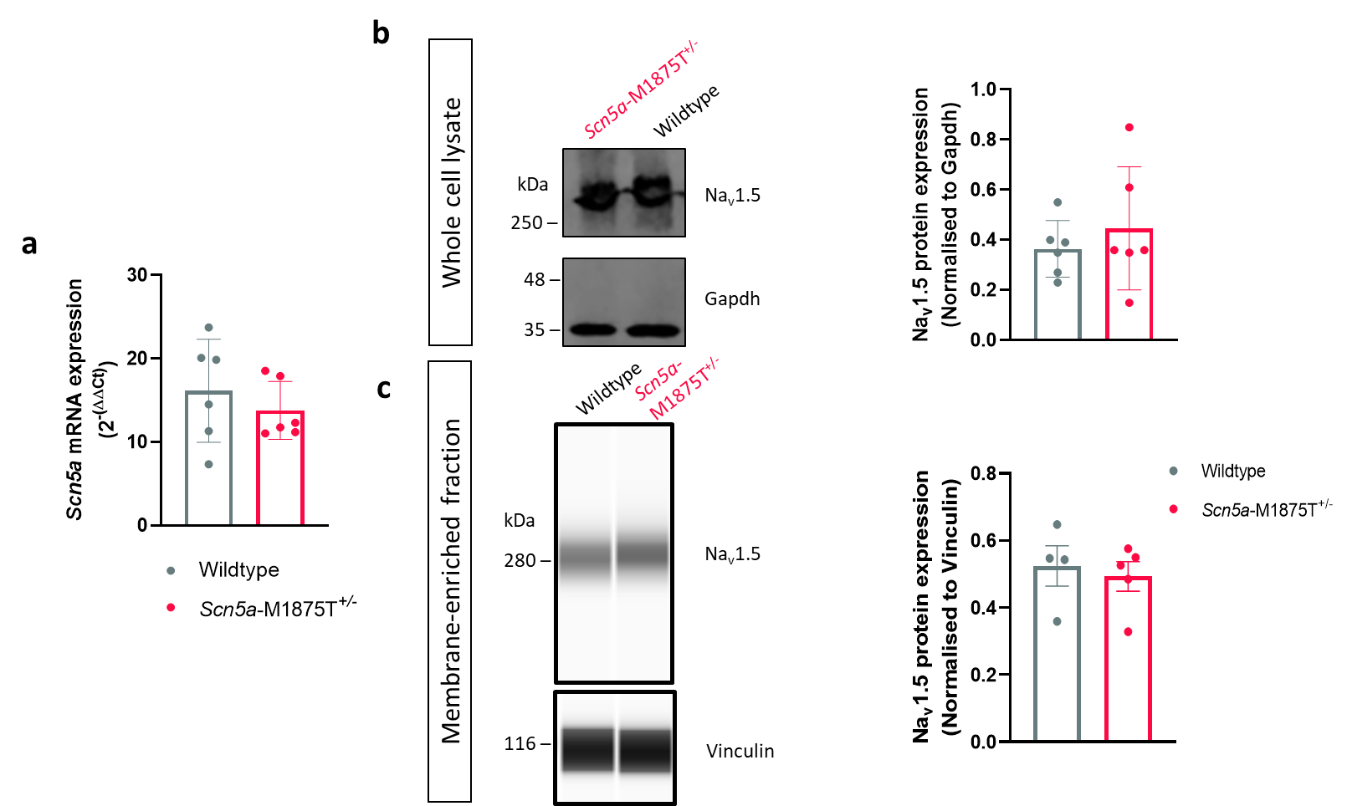


**Figure S4: Quantification of *Scn5a* mRNA and Na_v_1.5 protein expression in the *Scn5a*-M1875T^+/-^ and wildtype mouse hearts, when measured at the whole cell and isolated membrane fraction level. a)** *Scn5a* mRNA quantification of LA tissue samples from *Scn5a*-M1875T^+/-^ as well as WT mice measured using RT-qPCR. Expression levels were normalised against GAPDH (n=6 per group).

**b)** Na_v_1.5 protein expression levels in left atrial (LA) tissue samples from *Scn5a*-M1875T^+/-^ and wildtype mice were measured using western blotting. GAPDH was used as a loading control. A representative blot is shown (left) with band quantification (right) (n=6 per group). **c)** Na_v_1.5 protein quantification in membrane-enriched fractions of LA tissue samples from *Scn5a*-M1875T^+/-^ (n=4) and WT (n=5) mice using capillary-based automated western blotting. Vinculin was used as a loading control. The detected protein amount is visualised (left) and area under peak was quantified (right). There were no obvious changes between genotypes.


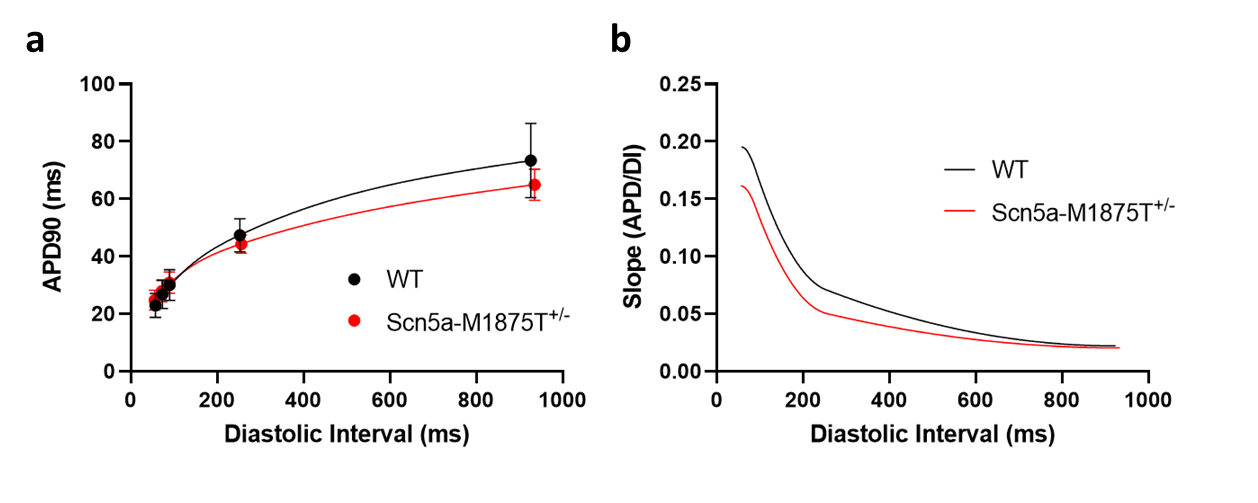


**Figure S5: APD restitution analysis from microelectrode experiments in left atria.**

Restitution curve (a) and derivative (b) of APD90 as a function of diastolic interval in WT and *Scn5a*-M1875T^+/-^ left atria illustrates a visually more pronounced effect of the M1875T mutation on APD slope at short diastolic intervals (short cycle lengths, high pacing frequencies).


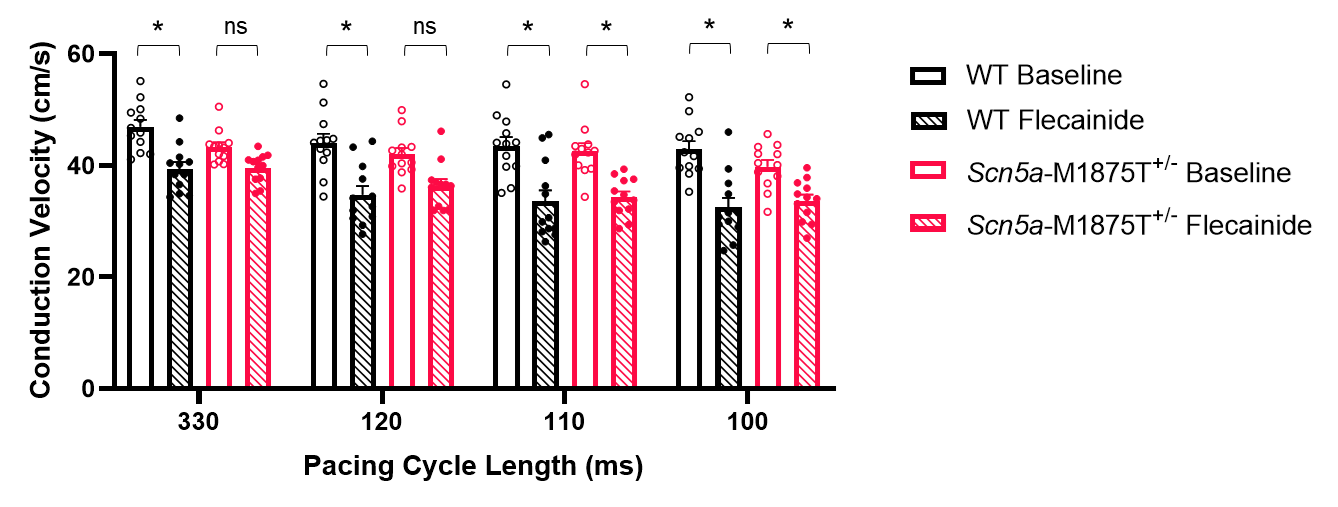


**Figure S6: Conduction velocity in left atria from optical mapping experiments at baseline and after addition of 1 µM flecainide at different pacing cycle lengths.** Means + SEM are shown. (12 atria per genotype group; ANOVA revealed no significant effect of genotype. ns non-significant; * p_adj_ < 0.05; Tukey-corrected post-hoc analysis)

This figure provides additional information to data presented in Figure 5 of the main manuscript.

**Supplemental Tables**


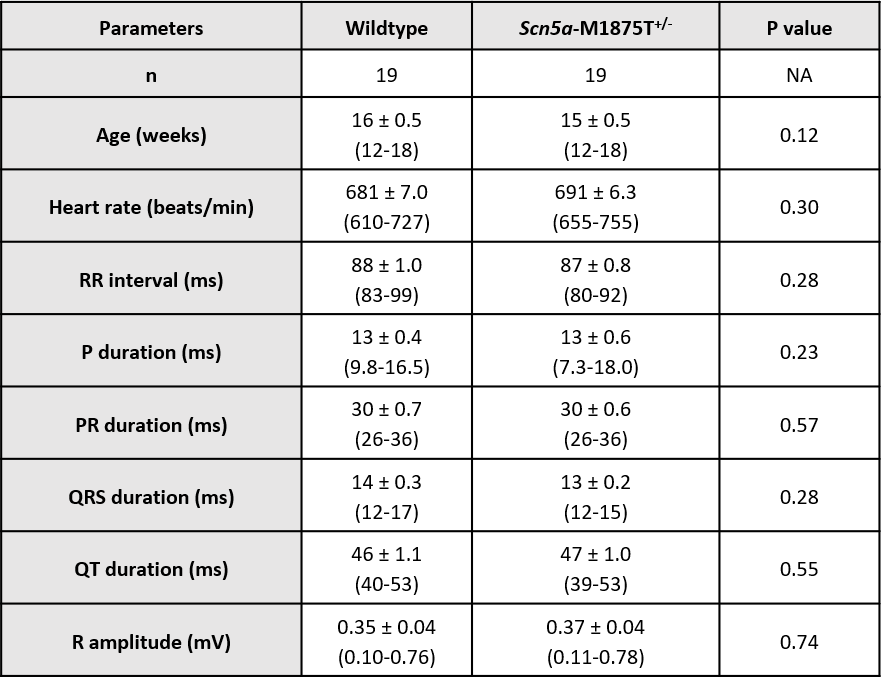


**Table S1:** **Electrocardiogram (ECG) characteristics of conscious wildtype and *Scn5a*-M1875T^+/-^** **mice.** Data are expressed as mean ± SEM (range).


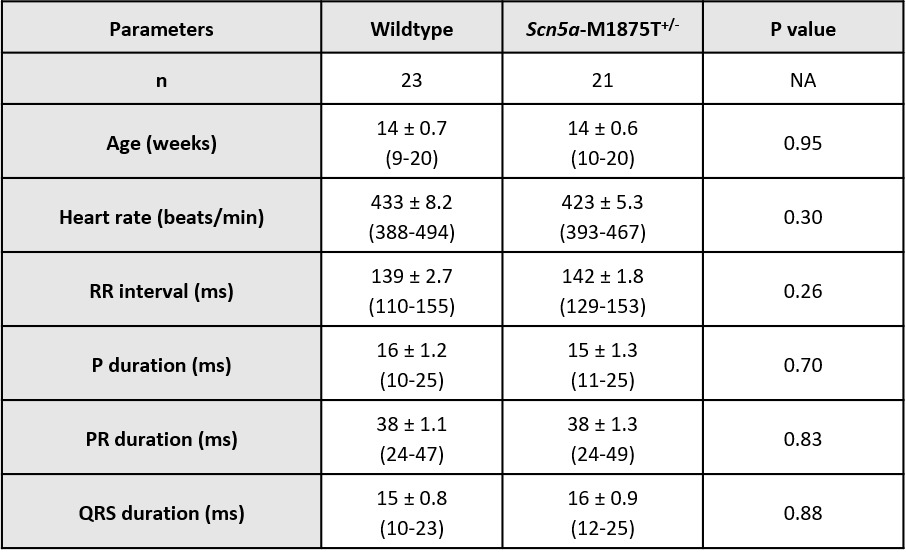


**Table S2:** **Electrocardiogram (ECG) characteristics of unconscious wildtype and *Scn5a*-M1875T^+/-^** **mice.** Data are expressed as mean ± SEM (range).


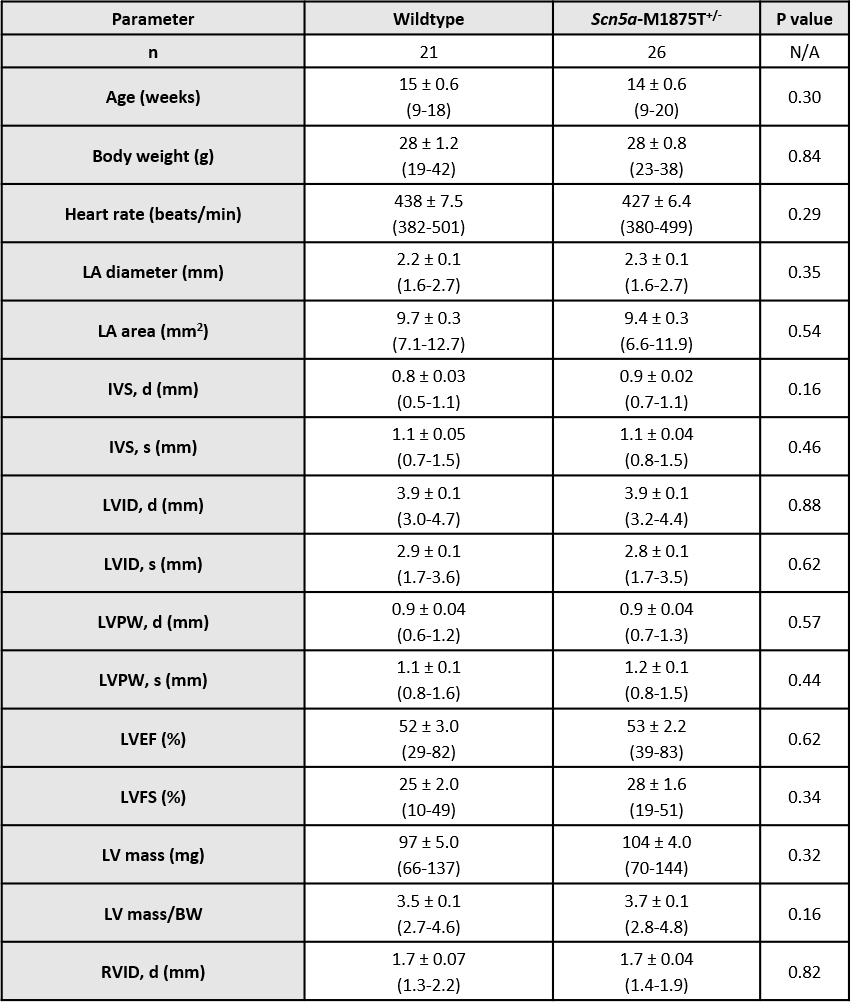


**Table S3:** **Echocardiographic measurements of wildtype and *Scn5a*-M1875T^+/-^** **mice.** Values are mean ± SEM (range). Mann-Whitney test used for statistical analysis; IVS, interventricular septum; LVID, left ventricular internal diameter; LVPW, left ventricular posterior wall; LVEF, left ventricular ejection fraction; LVFS, left ventricular fractional shortening; LV mass, left ventricular mass; BW, body weight: LA diameter, left atrial diameter; LA area, left atrial area; RVID, right ventricle internal diameter; d, diastole; s, systole.


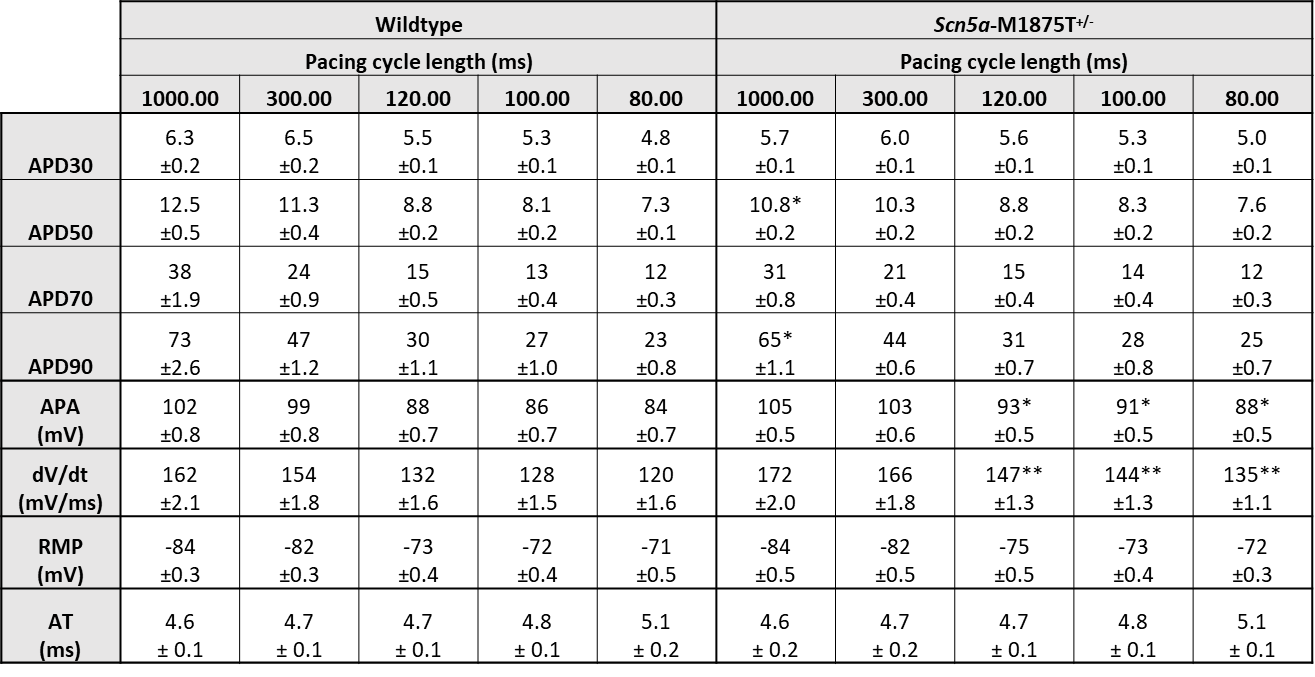


**Table S4: Action potential characteristics in wildtype and *Scn5a*-M1875T^+/-^** **left atria at all pacing cycle lengths tested using the sharp microelectrode technique.** Stated are means ± SEM for: action potential durations (APDs) at 30 (APD30), 50 (APD50), 70 (APD70) and 90% (APD90) repolarisation, action potential amplitude (APA), peak upstroke velocity (dV/dt), resting membrane potential (RMP) and activation times (ATs), at pacing cycle lengths (PCLs) of 80, 100, 120, 300 and 1000 ms. At 1000 ms PCL, APD50 and APD90 were significantly reduced in *Scn5a*-M1875T^+/-^ left atria relative to wildtype. APA was significantly larger in *Scn5a*-M1875T^+/-^ mutants at PCLs of 80-300 ms. dV/dt was faster in mutants when tested at PCLs of 80-120 ms. No significant changes in RMP or AT were detected at any PCL. Values are shown here for reference and as mean ± SEM. *p<0.05, **p<0.01, WT vs *Scn5a*-M1875T^+/-^, N=8 per group, n=24 WT, n=25 *Scn5a*-M1875T^+/-^).

**Extended methods**

# Generation of the *Scn5a*-M1875T construct and knock-in mouse model

## Targeting construct design:

The 3.7 kb genomic region, containing exon 28 of the *Scn5a* gene and right flanking region (RFR) was PCR amplified from mouse genomic DNA and (T-C) mutated using combination of subsequent PCR reactions with help of oligonucleotide pairs SCN5aA1d / SCN5aA1r, SCN5aA2d / SCN5aA2r and SCN5aMUT, subcloned and sequenced. The 5.1 kb left flanking region (LFR) containing exons 25-27, and intronic sequences was PCR amplified from mouse genomic DNA using oligonucleotides SCN5aBcld / SCN5aBclr and subcloned. The 1.3 kb genomic region, containing exon 24 and intronic sequences was added to the LFR. All individual clones were verified by sequencing and assembled into the final targeting construct (pSCN5a_targ3) in the order depicted in Figure 1a. The pBluescript based plasmid backbone together with the negative selection marker (thymidine kinase cassette) were added to the left flanking region (not shown). The 1.9 kb DNA fragment, containing a positive selection marker (neomycin cassette flanked by two LoxP sites) was cloned between LFR and RFR.

## Southern blot DNA probes cloning:

The 1.4 kb HR probe was PCR amplified from mouse genomic DNA using oligonucleotides SCN5aHRd2 and SCN5aHRr2, subcloned and sequenced. The So1, So2, So3, So4 and So5 probes were PCR amplified from mouse genomic DNA, subcloned and sequenced using pairs of oligonucleotides: SCN_SO1D/SCN_SO1R, SCN_SO2D/SCN_SO2R, SCN_SO3D/SCN_SO3R, SCN_SO4D/SCN_SO4R, and SCN_SO5D/SCN_SO5R accordingly.

## Scn5a exon 28 specific gRNA’s selection and cloning for the CRISPR/cas9 system:

Three gRNA targets were selected in a close proximity to the mutation site. These gRNAs were cloned in plasmid gRNA_Cloning Vector (gift from George Church (Addgene plasmid # 41824))^1^ digested with the *Afl*II restriction endonuclease, using the Gibson assembly methods with help of oligonucleotide pairs: SCN5a_ Insert_F/SCN5a_ Insert_R, SCN5a_ Insert_F2/SCN5a_ Insert_R2, and SCN5a_Insert_F3/SCN5a_Insert_R3 resulting in plasmids pgRNA_SCN1, pgRNA_SCN2, and pgRNA_SCN3 accordingly.

## ES cell transfection and selection of targeted clones

CV19 ES cells (passage 13 [129Sv x C57BL/6J]) were expanded in HEPES-buffered Dulbecco’s modified Eagle’s medium supplemented with 15% fetal bovine serum (PAA), nonessential amino acids, L‑glutamine, β-mercaptoethanol, 1,000 U per mL of recombinant leukemia inhibitory factor (LIF) (MERCK Millipore), and antibiotics (penicillin [100 U/ml] and streptomycin [100 µg/ml]). For electroporation, 2 x 10^7^ cells were resuspended in 0.8 ml Capecchi buffer (20 mM HEPES [pH 7.4], 173 mM NaCl, 5 mM KCl, 0.7 mM Na_2_HPO_4_, 6 mM dextrose, 0.1 mM β-mercaptoethanol)^2^. The targeting vector DNA pSCN5a_targ3 (100 µg) was electroporated together with 70 µg of each plasmid DNA’s: pgRNA_SCN1, pgRNA_SCN2, pgRNA_SCN3 and hCAS9 (cas9 coding plasmid was a gift from George Church (Addgene plasmid # 41815))^1^ at 25 µF and 400V in 0.8 mm electroporation cuvettes (Gene Pulser; Bio-Rad). After electroporation, cells were cultivated for 10 min at room temperature and plated onto ten 100-mm diameter culture dishes containing a gamma-irradiated monolayer of mouse primary G418-resistant fibroblast feeder cells. Thirty-two hours later, 350 µg of G418 (Invitrogen) per mL and 0.2 μM 2’-deoxy-2’-fluoro-β-D-arabinofuranosyl-5-iodouracil (FIAU) (Moravek Biochemicals and Radiochemicals, USA) were added to the culture medium. The medium was replaced every day, and colonies were picked and analysed 8 days after plating.

## DNA Southern blot analysis

Positively targeted ES cell clones were analyzed using the Southern-blot DNA method. Approximately 5-10 µg of genomic DNA was digested with *Eco*RI, fractionated on 0.8% agarose gels, and transferred to GeneScreen nylon membranes (NEN DuPont). The membranes were hybridized with a ^32^P-labeled 1.4 kb HR probe and washed with (final concentrations) 0.5x SSPE (1x SSPE is 0.18 M NaCl, 10 mM NaH_2_PO_4_, and 1mM EDTA [pH 7.7]) and 0.5% sodium dodecyl sulfate at 65°C. After first screening, correctly targeted clones were proven using the *Bam*HI and additionally *Hind*III digestion, using a ^32^P‑labeled 1.8 kb probe So3 containing internal sequences to the targeted homology. The Southern blot analysis of DNA samples isolated from F1 mouse tail biopsy using probe So3 is presented on (Figure 1b).

## Blastocyst injection

Correctly targeted ES cells from 9C clone were injected into 3.5-day B6D2F1 blastocysts. Routinely, we are injecting 12 to 14 ES cells into one blastocoele. After injection, blastocysts were kept in KSOM medium and subsequently transferred into the uteri of 2.5-day pseudopregnant CD-1 foster mice. The mice carried pups to term. Chimeras were identified by their agouti coat colour contribution. For the germ-line transmission high percentage male chimaeras were crossed to the C57BL/6J female mice. Heterozygous agouti offsprings (*Scn5a*-M1875^+/-^) were confirmed by Southern blot analysis (Figure 1b), and the mutated part of exon 28 was PCR amplified using primers pair SCN5a_Sequenc_F/ SCN5a_Sequenc_R and sequenced (Figure 1c and Supplementary Figure 1a). Mice were kept in specific pathogen-free animal facilities.

## Deletion of the neo cassette

The deletion of the Neo cassette (Figure 1a) was performed by crossing mice with the total CRE deleter transgenic animals and verified by PCR analysis and further confirmed by the Southern blot analysis (not shown), using different probes, including the probe containing the Neo cassette sequences.

## List of oligonucleotides used in the study:

SCN5aA1d *TGATATC*GTGAGGAGCTGGAAGCCTTGAG

SCN5aA1r CCGTCTGGATCTTCAGGGCATCCATCTC

SCN5aA2d TGAAGATCCAGACGGAGGAGAAATTCATGG

SCN5aA2r *TGCGGCCGC*ATTTATACACGAAGCTTAGTGAGAAGTG

SCN5aMUT GAGATGGATGCCCTGAAGATCCAGACGGAGGAGAAATTCATGG

SCN5aBcld *TGTCGAC*TGCATGCACTTGATGGCCTCAC

SCN5aBclr *TGAATTC*CCTAGTCCATCTCCTCCTAACA

SCN5aHRd2 GACTAAGTGATGCTAAAACACAT

SCN5aHRr2 TGTGTGTATGTGTAGAGGTTGTC

SCN_SO1D *CAGTCTCAAGCGTCTCTTGGAGTCGA*CCTCACTACAGCCGCACACTCAC

SCN_SO1R *CTGCTCTAGACGTCTCTGAGAGTCGA*CCAAGAAAGTCAGGCTAGCAGAGAG

SCN_SO2D *CAGTCTCAAGCGTCTCTTGGAGTCGAC*AGAATAAATAACATCTACTCCATAAG

SCN_SO2R *CTGCTCTAGACGTCTCTGAGAGTCGAC*AAGTCAAGGAAGACATAGCAG

SCN_SO3D *CAGTCTCAAGCGTCTCTTGGAGTCGA*CTAGGCTGATGCAGTGCTGAAG

SCN_SO3R *CTGCTCTAGACGTCTCTGAGAGTCGAC*ACATGTACAGTGTGTTTCCCGTT

SCN_SO4D *CAGTCTCAAGCGTCTCTTGGAGTCGAC*TCCTGCTGGCTTTTGATTGTGC

SCN_SO4R *CTGCTCTAGACGTCTCTGAGAGTCGAC*AAAAGGGACATCTCTTGGGAAACT

SCN_SO5D *CAGTCTCAAGCGTCTCTTGGAGTCGA*CAGAAAACTTGAGCCAATCCAC

SCN_SO5R *CTGCTCTAGACGTCTCTGAGAGTCGA*CCCCGTGGGCACCGTTTAG

SCN5a_ Insert_F TTTCTTGGCTTTATATATCTTGTGGAAAGGACGAAACACCGGCCCTGAAGATCCAGATGG

SCN5a_ Insert_R GACTAGCCTTATTTTAACTTGCTATTTCTAGCTCTAAAACCCATCTGGATCTTCAGGGCC

SCN5a _Insert_F2 TTTCTTGGCTTTATATATCTTGTGGAAAGGACGAAACACCGCTCGGGGAGTCTGGGGAGA

SCN5a _Insert_R2 GACTAGCCTTATTTTAACTTGCTATTTCTAGCTCTAAAACTCTCCCCAGACTCCCCGAGC

SCN5a _Insert_F3 TTTCTTGGCTTTATATATCTTGTGGAAAGGACGAAACACCGTAGGAGATCTTGGAAGGAT

SCN5a _Insert_R3 GACTAGCCTTATTTTAACTTGCTATTTCTAGCTCTAAAACATCCTTCCAAGATCTCCTAC

SCN5a_Sequenc_F GCCCTGTCCGACTTTGCCGAT

SCN5a_Sequenc_R CTGGCGGAAGAGGAAGGAAGCAT

Sequences used for cloning are italicized. Scn5a-M1875T mutated nucleotide is underlined.

# mRNA and protein expression levels

## **RNA preparation and quantitative real-time PCR**

RNA was purified from LA and left ventricle tissue as previously described ^3^. Briefly, LA and left ventricle tissue was homogenised using the Precellys homogeniser (Precellys) and the RNA was purified using the RNEasy mini kit (Qiagen). Total RNA (1 µg) was used as a template for reverse transcription with the High-Capacity cDNA Reverse Transcription kit (Thermo Fisher) according to the manufacturer’s protocol. A TaqMan® probe to *Scn5a* (Mm01342518_m1; Applied Biosystems) was used to quantify mRNA levels by RT-PCR using TaqMan^TM^ Universal PCR Master Mix. *Gapdh* (Mm99999915_g1; Applied Biosystems) was used for normalisation. The results of six separate experiments, each calculated from three technical replicates, were pooled using the ΔΔCt method.

## Western blotting methods

Western blotting of whole LA, RA and LV protein lysate was carried out as previously described ^3^. Briefly, snap-frozen cardiac chambers were lysed in ice-cold radioimmune precipitation buffer (Sigma) with Halt Protease and Phosphatase Inhibitor mixture (Thermo Fisher) using a mechanical Precellys 24 tissue homogeniser (Precellys) or cryogrinding. Total protein concentration was quantified using the DC Protein Assay kit (Bio-Rad). After normalisation of protein concentration using SDS reducing sample buffer, samples were denatured at 95°C for 5 minutes before being resolved by SDS-PAGE and immunoblotted using primary antibodies directed against Nav1.5 (D9J7S; Cell Signaling) or pro-ANP (ab180649, Abcam). Quantification of GAPDH protein expression (14C10; Cell Signaling) served as a loading control. Protein-antibody complexes were visualised using HRP-based chemiluminescence.

To collect a membrane enriched LA protein lysate, the LA was homogenised in ice cold lysis buffer (50 mM Tris-Base (pH 7.5), 2 mM EDTA, 5 mM EGTA, 5 mM DTT, 0.05% Digitonin) supplemented with Halt Protease and Phosphatase Inhibitor mixture. Cytoplasmic fraction was collected through centrifugation at 17,000 g for 30 minutes at 4°C. The resulting pellet was resuspended in ice cold lysis buffer supplemented with 1% Triton-X before repeating centrifugation. The resulting supernatant was defined as a membrane-enriched fraction. Protein concentration was determined using DC protein assay (Bio-Rad). Na_v_1.5 protein expression was determined in the membrane fraction using Western capillary electrophoresis (WES) method (ProteinSimple, San Jose, CA). Left atrial membrane fractions from wild-type mice and mice possessing the mutation were loaded into WES 13-well plates for separation using capillary electrophoresis (66-440 kDa) following manufacturer’s instructions. Antibodies directed against Na_v_1.5 (Cell Signalling) and Vinculin (Cell signalling) were both used at a dilution of 1:50 in antibody diluent (ProteinSimple). The relative amount of each protein was analysed through the areas under peaks from the chemiluminescence chromatograms by Compass for SW software (ProteinSimple).

# ECG recordings *in vivo*

Non-invasive electrocardiograms (ECG) were recorded in conscious young adult mice (8-19 weeks) using a tunnel system for gentle restraint (ecgTunnel, EMKA Technologies, Paris, France) ^4^. ECG recordings of 3-6 minutes were analysed using ECGauto software (EMKA Technologies, Paris, France). by constructing 3-5 average signals from 20 consecutive beats each. ECGs were also recorded in isoflurane-sedated mice during echocardiography as below.

# Echocardiography *in vivo*

Transthoracic echocardiography was performed in sedated mice (0.5-2% isoflurane, supplemented with 100% O_2_) kept on a heated platform using the Vevo® 2100 system (VisualSonics, Amsterdam, Netherlands) as reported previously ^5^. In order to improve image quality, the thorax of the mice was shaved and remaining hair removed using depilatory cream. Heart rate was maintained at 450 ± 70 bpm.

Left atria (LA) were visualised in the parasternal long axis view in the plane of the aortic root. LA area and diameter were measured during pre-atrial contraction, using the P-wave of the limb ECG trace as a guide.

# Histological analysis

Hearts were fixed in formalin and paraffin-embedded tissues were cut into slices of 4 µm. Sections were dewaxed, stained with hematoxylin and eosin for overviews and subsequently dehydrated, embedded and imaged on a NanoZoomer 2.0-HAT (Hamamatsu).

Slides used for quantitative analysis were cooked in citrate buffer for antigen retrieval. Autofluorescence was quenched with a 0.25% Sudan black solution for 30 min and samples were blocked with 2% BSA/2.2% Glycine for 1 h at room temperature (RT). Wheat germ agglutinin (WGA) with Alexa Fluor™ 488 Conjugate (1:200, W11261, Invitrogen) was applied for 2 h at RT.

Images of WGA-stained cardiac tissue were obtained with a confocal microscope equipped with an Aurox Clarity (Aurox Ltd.) spinning disc unit and a 20x EC Plan-Neofluar objective (420353-9900-000, Zeiss, NA=0.5) run with Aurox Visionary (Aurox Ltd.) software.

Quantitative analysis of WGA-stained area and cardiac cell diameters was carried out using a published ImageJ plugin for atrial histological analysis (JavaCyte^6^), with minor adjustments.

# Atrial cardiomyocyte isolation

Murine hearts were excised under deep terminal anaesthesia (4% isoflurane inhalation in O_2_, 1.5 L/min) and perfused at 4 mL.min^-1^ at 37°C on a vertical Langendorff apparatus with the following solutions, equilibrated with 100% O_2_: (i) HEPES-buffered, Ca^2+^-free, modified Tyrode’s solution containing in mM: NaCl 145, KCl 5.4, MgSO_4_ 0.83, Na_2_HPO_4_ 0.33, HEPES 5, and glucose 11 (pH 7.4, NaOH) x 5 min; (ii) Tyrode’s enzyme solution containing 640 µg/mL collagenase type II (270 U/mg), 600 µg/ml collagenase type IV (270 U/mg) and 50 µg/mL protease (Worthington, Lakewood, NJ), 20 mM taurine and 3 μM CaCl_2_ × 8-12 min. The heart was removed from the Langendorff setup and perfused with 5 mL of modified Kraftbruhe (KB) solution containing in mM: DL-potassium aspartate 10, L-potassium glutamate 100, KCl 25, KH_2_PO_4_ 10, MgSO_4_ 2, taurine 20, creatine 5, EGTA 0.5, HEPES 5, 0.1% BSA, and glucose 20 (pH 7.2, KOH).

The LA was dissected free and cardiomyocytes were dissociated gently with fire-polished glass pipettes (2 to 1 mm diameter in sequence). Cells were re-suspended in 2 mL KB buffer and Ca^2+^ was gradually reintroduced to the cell suspension incrementally over a period of 2 hours to reach a final concentration of 1 mM. All experiments were performed within 8 hours of isolation.

# Whole-cell patch clamp electrophysiology of isolated atrial cardiomyocytes

Dissociated murine LA cardiomyocytes were plated on, and allowed to adhere to, laminin-coated coverslips (10 mm diameter) for at least 20 minutes. Coverslips were transferred to a recording chamber and were continually superfused at 3 mL.min^-1^, with a low Na^+^ external solution containing in mM; NaCl 10, KCl 4.5, C_5_H_14_CINO 130, CaCl_2_ 1, MgCl_2_ 1.2, HEPES 10 and glucose 10 (pH 7.4 with CsOH). To block L-type Ca^2+^ currents, 2 mM NiCl_2_ was added to the superfusate. Experiments were performed at 22 ± 0.5°C. Whole-cell patch clamp recordings were obtained in voltage-clamp mode using borosilicate glass pipettes (tip resistances 1.5-3 MΩ) .

For Na^+^ current recordings, the pipette solution contained in mM: CsCl 115, NaCl 5, EGTA 10, HEPES 10, MgATP 5, TEACl 20 and MgCl_2_ 0.5 (pH 7.2, KOH). Voltage-dependent Na^+^ currents were evoked by 5 mV step depolarisations (100 ms) from a holding potential of -100 mV to test potentials ranging from -95 mV to +40 mV. Cells were excluded from analysis if there was no reversal of the sodium current by +40mV. To investigate Na_v_1.5 voltage-dependent inactivation kinetics, cells were subject to 500 ms pre-pulses ranging from -120 mV to -40 mV, followed by a 100 ms step to -30 mV. For Na_v_1.5 time-dependent recovery kinetics, a standard two pulse protocol was used (-120 mV to -30 mV, 20 ms), with the time between the two pulses incrementally varying between 5 and 950 ms.

All recordings and analysis protocols were performed using an Axopatch 200B amplifier (Molecular Devices, USA) and digitized at 50 kHz using a CED micro1401 driven by Signal v6 software (Cambridge Electronic Design, Cambridge, UK). Series resistance was compensated, ranging between 60-100% for all cells. Experiments were terminated if series resistance abruptly changed or was above 10 MΩ.

# Atrial microelectrode recordings

Our investigations focus on the left atrium (LA). This is mostly due to practical reasons, e.g. less spontaneous activity in the left compared to the right atrium.

As previously described ^7-9^, following isolation the LA was immediately transferred into a dissecting chamber and continuously superfused at 10 mL.min^-1^ with a bicarbonate buffered Krebs-Henseleit (KH) solution containing in mM: NaCl 118; NaHCO_3_ 24.88; KH_2_PO_4_ 1.18; Glucose 11; MgSO_4_ 0.83; CaCl_2_ 1.8; KCl 3.52, equilibrated with 95% O_2_/5% CO_2_, 36-37°C, pH 7.4. Micro-dissection and pinning out of the LA was performed using a dissection microscope (Stemi SV 11, Zeiss, Germany). The LA was paced at 1–10 Hz via bipolar platinum electrodes. Action potentials (APs) were recorded from freely contracting LA using custom made glass floating microelectrodes containing 3 M KCl, (resistance 15-30 MΩ). Voltage signals were amplified and digitised at 20 kHz and were unfiltered (Axoclamp 2B; Molecular Devices, California, USA; Spike2 software Cambridge Electronic Design, Cambridge, UK). Measured parameters included the resting membrane potential (RMP), action potential amplitude (APA), peak depolarisation rate (dV/dt) and action potential duration (APD) at 30-90% repolarisation. APs were only analysed following sufficient rate adaptation achieved after at least 50 stimulated APs at each frequency.

# Atrial optical mapping

Optical mapping of the LA was conducted as previously described ^10, 11^. Isolated whole hearts were loaded on to a vertical Langendorff apparatus and perfused with a standard KH solution. Hearts were perfused at 4 mL.min^-1^ (equilibrated with 95%O_2_/5%CO_2_ and heated to 36-37°C, pH 7.4). Hearts were loaded with 25 µL of voltage sensitive dye Di-4-ANEPPS at a concentration of 5 mg/mL, diluted in 1 mL of KH solution and delivered via bolus port injection over 3-5 minutes. The LA was then isolated and pinned in a superfusion chamber containing 37°C KH solution for transfer to the optical mapping setup, anterior surface facing up.

In the optical mapping system, atria were superfused with KH solution (95%O_2_/5%CO_2_, 36-37°C) containing contraction uncoupler Blebbistatin (35 µM). For imaging, atria were illuminated by two dual LEDs at 530 nm. A 630 nm long-pass ﬁlter was used to separate emitted ﬂuorescence, imaged using an ORCA ﬂash 4.0 CMOS camera (Hamamatsu, Japan). Images were acquired at a framerate 0.987 kHz and pixel size of 71 µm/pixel^2^. Atria were paced using bipolar platinum electrodes delivering 2 ms pulses at twice diastolic threshold (minimum voltage required to elicit APs).

One-minute baseline recording was taken following a 10 minute equilibration period to ensure contraction uncoupling and temperature re-stabilisation. During imaging, atria were initially paced at 330 ms pacing cycle length (PCL). A ‘ramp’ pacing protocol was then initiated, in which the atria were paced at 120 ms PCL for 100 stimuli and then PCL was reduced from 120 ms to 80 ms in 10 ms intervals every 20 stimuli. After taking baseline recordings, LED illumination was switched off and the superfusion solution replaced with an identical solution containing flecainide, a clinically-used sodium channel blocker^12^ at a concentration of 1 µM and then 5 µM (or control solution without flecainide for time control experiments).

Subsequent recordings were then made as described above after 20 minutes superfusion with 1 µM flecainide solution, and then further 15 minutes with 5 µM flecainide solution. Atria were paced at 330 ms PCL continuously in dark conditions between recordings.

From these recordings, APD and conduction velocity (CV) were mapped across the LA using ElectroMap software ^11^. Atria were removed from analysis at a given PCL if loss of 1:1 capture ratio with pacing stimuli (i.e. missed beats) was observed.

**Supplemental References**

[1] Mali P, Yang L, Esvelt KM, Aach J, Guell M, DiCarlo JE, et al. RNA-guided human genome engineering via Cas9. *Science (New York, NY)* 2013; **339**: 823-826.

[2] Thomas KR, Capecchi MR. Site-directed mutagenesis by gene targeting in mouse embryo-derived stem cells. *Cell* 1987; **51**: 503-512.

[3] Reyat JS, Chua W, Cardoso VR, Witten A, Kastner PM, Kabir SN, et al. Reduced left atrial cardiomyocyte PITX2 and elevated circulating BMP10 predict atrial fibrillation after ablation. *JCI Insight* 2020; **5**.

[4] Silbernagel N, Walecki M, Schafer MK, Kessler M, Zobeiri M, Rinne S, et al. The VAMP-associated protein VAPB is required for cardiac and neuronal pacemaker channel function. *FASEB J* 2018; **32**: 6159-6173.

[5] Blana A, Kaese S, Fortmüller L, Laakmann S, Damke D, van Bragt K, et al. Knock-in gain-of-function sodium channel mutation prolongs atrial action potentials and alters atrial vulnerability. *Heart Rhythm* 2010; **7**: 1862-1869.

[6] Winters J, von Braunmuhl ME, Zeemering S, Gilbers M, Brink TT, Scaf B, et al. JavaCyte, a novel open-source tool for automated quantification of key hallmarks of cardiac structural remodeling. *Scientific reports* 2020; **10**: 20074.

[7] Holmes AP, Yu TY, Tull S, Syeda F, Kuhlmann SM, O'Brien SM, et al. A Regional Reduction in Ito and IKACh in the Murine Posterior Left Atrial Myocardium Is Associated with Action Potential Prolongation and Increased Ectopic Activity. *PLoS One* 2016; **11**: e0154077.

[8] Syeda F, Holmes AP, Yu TY, Tull S, Kuhlmann SM, Pavlovic D, et al. PITX2 Modulates Atrial Membrane Potential and the Antiarrhythmic Effects of Sodium-Channel Blockers. *J Am Coll Cardiol* 2016; **68**: 1881-1894.

[9] Lemoine MD, Duverger JE, Naud P, Chartier D, Qi XY, Comtois P, et al. Arrhythmogenic left atrial cellular electrophysiology in a murine genetic long QT syndrome model. *Cardiovascular research* 2011; **92**: 67-74.

[10] Yu TY, Syeda F, Holmes AP, Osborne B, Dehghani H, Brain KL, et al. An automated system using spatial oversampling for optical mapping in murine atria. Development and validation with monophasic and transmembrane action potentials. *Prog Biophys Mol Biol* 2014; **115**: 340-348.

[11] O'Shea C, Holmes AP, Yu TY, Winter J, Wells SP, Correia J, et al. ElectroMap: High-throughput open-source software for analysis and mapping of cardiac electrophysiology. *Sci Rep* 2019; **9**: 1389.

[12] Ramos E, O'Leary M E. State-dependent trapping of flecainide in the cardiac sodium channel. *J Physiol* 2004; **560**: 37-49.
